# The Vessels-Bone Axis: Iliac Artery Calcifications, Vertebral Fractures and Vitamin K from VIKI Study

**DOI:** 10.3390/nu13103567

**Published:** 2021-10-12

**Authors:** Maria Fusaro, Giovanni Tripepi, Mario Plebani, Cristina Politi, Andrea Aghi, Fulvia Taddei, Enrico Schileo, Martina Zaninotto, Gaetano La Manna, Giuseppe Cianciolo, Maurizio Gallieni, Laura Cosmai, Piergiorgio Messa, Maura Ravera, Thomas L. Nickolas, Serge Ferrari, Markus Ketteler, Giorgio Iervasi, Maria Cristina Mereu, Roberto Vettor, Sandro Giannini, Lorenzo Gasperoni, Stefania Sella, Maria Luisa Brandi, Luisella Cianferotti, Raffaele De Caterina

**Affiliations:** 1 National Research Council (CNR), Institute of Clinical Physiology (IFC), 56124 Pisa, Italy; 2 Department of Medicine, University of Padova, 35128 Padova, Italy; iervasi@ifc.cnr.it (G.I.); roberto.vettor@unipd.it (R.V.); 3 CNR-IFC, Clinical Epidemiology of Renal Diseases and Hypertension, Ospedali Riuniti, 89124 Reggio Calabria, Italy; gtripepi@ifc.cnr.it (G.T.); politicristina89@gmail.com (C.P.); 4 Laboratory Medicine Unit, Department of Medicine, University of Padua, 35129 Padua, Italy; mario.plebani@unipd.it (M.P.); martina.zaninotto@aopd.veneto.it (M.Z.); 5 Clinica Medica 1, Department of Medicine, University of Padua, 35128 Padua, Italy; andrea.aghi@gmail.com (A.A.); sandro.giannini@unipd.it (S.G.); stefania.sella@unipd.it (S.S.); 6 Bioengineering and Computing Laboratory, IRCCS Istituto Ortopedico Rizzoli, 40136 Bologna, Italy; fulvia.taddei@ior.it (F.T.); enrico.schileo@ior.it (E.S.); 7 Nephrology, Dialysis and Renal Transplant Unit, IRCCS—Azienda Ospedaliero-Universitaria di Bologna, Alma Mater Studiorum University of Bologna, 40126 Bologna, Italy; gaetano.lamanna@unibo.it (G.L.M.); giuseppe.cianciolo@aosp.bo.it (G.C.); lorenzo.gasperoni3@gmail.com (L.G.); 8 Department of Biomedical and Clinical Sciences ‘Luigi Sacco’, Università di Milano, 20157 Milano, Italy; maurizio.gallieni@unimi.it; 9 Nephrology Unit, ASST Fate Bene Fratelli Sacco, 20157 Milano, Italy; lacos@iol.it; 10 Unit of Nephrology, Dialysis and Kidney Transplantation, Fondazione IRCCS Ca’ Granda Ospedale Maggiore Policlinico di Milano, 20157 Milan, Italy; piergiorgio.messa@policlinico.mi.it; 11 Department of Clinical Sciences and Community Health, University of Milan, 20157 Milan, Italy; 12 Policlinico San Martino, 16132 Genova, Italy; maura.ravera@hsanmartino.it; 13 Division of Nephrology, Department of Medicine, Columbia University Irving Medical Center, New York, NY 10032, USA; tln2001@cumc.columbia.edu; 14 Service des Maladies Osseuses, Département de Médecine, HUG, 1205 Genève, Switzerland; serge.ferrari@unige.ch; 15 Department of General Internal Medicine and Nephrology, Robert-Bosch-Krankenhaus, 70376 Stuttgart, Germany; Markus.Ketteler@rbk.de; 16 Independent Researcher, 09100 Cagliari, Italy; crissmer5412@gmail.com; 17 Department of Surgery and Translational Medicine, University of Florence, Viale Pieraccini 6, 50139 Florence, Italy; marialuisa.brandi@unifi.it (M.L.B.); luisella.cianferotti@unifi.it (L.C.); 18 Cardiology, Cardiovascular Division, Pisa University Hospital, University of Pisa, Via Paradisa 2, 56126 Pisa, Italy; raffaele.decaterina@unipi.it; 19 Fondazione Villa Serena per la Ricerca, Città Sant’Angelo, 65013 Pescara, Italy

**Keywords:** peripheral vascular disease, epidemiology, metabolic syndrome, vitamin K

## Abstract

Vascular calcification and fragility fractures are associated with high morbidity and mortality, especially in end-stage renal disease. We evaluated the relationship of iliac arteries calcifications (IACs) and abdominal aortic calcifications (AACs) with the risk for vertebral fractures (VFs) in hemodialysis patients. The VIKI study was a multicenter cross-sectional study involving 387 hemodialysis patients. The biochemical data included bone health markers, such as vitamin K levels, vitamin K-dependent proteins, vitamin 25(OH)D, alkaline phosphatase, parathormone, calcium, and phosphate. VF, IACs and AACs was determined through standardized spine radiograms. VF was defined as >20% reduction of vertebral body height, and VC were quantified by measuring the length of calcium deposits along the arteries. The prevalence of IACs and AACs were 56.1% and 80.6%, respectively. After adjusting for confounding variables, the presence of IACs was associated with 73% higher odds of VF (*p* = 0.028), whereas we found no association (*p* = 0.294) for AACs. IACs were associated with VF irrespective of calcification severity. Patients with IACs had lower levels of vitamin K2 and menaquinone 7 (0.99 vs. 1.15 ng/mL; *p* = 0.003), and this deficiency became greater with adjustment for triglycerides (0.57 vs. 0.87 ng/mL; *p* < 0.001). IACs, regardless of their extent, are a clinically relevant risk factor for VFs. The association is enhanced by adjusting for vitamin K, a main player in bone and vascular health. To our knowledge these results are the first in the literature. Prospective studies are needed to confirm these findings both in chronic kidney disease and in the general population.

## 1. Introduction

Vertebral fractures (VF) are extremely common in the general population and are associated with skeletal fragility. According to a recent meta-analysis, approximately 25% of all postmenopausal women develop a VF during their lifetime, with the prevalence of this condition increasing with age [1]. Notably, 28% of VF are asymptomatic [1], and their diagnosis can be missed in up to a half of cases [2,3,4], thus hindering the identification of patients at an increased risk of further vertebral and non-vertebral fractures, also known as VF cascade events [5]. The mineral and bone disorders (MBD) related to chronic kidney disease (CKD) make patients with renal insufficiency exposed to fractures much more than the general population [6].This is particularly true for hemodialysis patients. A previous study by our group [7] found VF prevalence to exceed 50% in hemodialysis patients (55%), similar to that of a control group affected by primary osteoporosis, and similar to the 50% prevalence reported for osteopenic postmenopausal women [4]. A similarly high prevalence of VF (57%) has also been reported in a renal transplant cohort [8]. Detection of VF is particularly relevant in hemodialysis patients because VF, just as with vascular calcifications (VC) [9], associate with an increased risk of cardiovascular disease (CVD) [10]. VC and VF also associate both in the general aging population [11] and in hemodialysis patients [7].The link between VF and VC is described as bidirectional [12,13], so that the concept of a bone-vascular axis is becoming common in CKD research [14,15].

Vitamin K is a known regulator of bone mineralization and VC, and is essential for the synthesis of some vitamin K-dependent proteins (VKDPs). These include osteocalcin or bone Gla protein (BGP) and matrix Gla protein (MGP) [16]. Vitamin K exists in different forms, called K-vitamers, including vitamin K1 (phylloquinone, PK), found mainly in green leafy vegetables and fruits; and vitamin K2 (menaquinones, MKs), which comprises various K-vitamers, named MK4-13 [17], synthesized by intestinal bacteria or found in cheese, meat and fermented soya derivatives [16,18]. In the Vitamin K Italian (VIKI) dialysis Study [19], we found that vitamin K1 deficiency had the strongest association with VF, whereas deficiency of MK4 and MK7 was most strongly associated with VC of the aortic and iliac arteries, respectively. This finding has drawn attention to the calcification of iliac arteries. There is indeed growing evidence that VC can be an indicator of adverse cardiovascular and bone clinical outcomes in CKD patients [20]. Iliac arteries, being predominantly, but not exclusively, muscular, might be a good target of diagnostic investigations to detect medial and intimal calcifications, so they have been included in the Adragao calcification score [21], which performed better than aortic calcifications in predicting all-cause and cardiovascular mortality in non-hemodialysis CKD patients [22]. However, iliac artery calcifications (IACs) have been rarely related to bone outcomes, and the most recent studies that attempted to associate VC to VF [23] have focused their interest on abdominal aorta calcifications (AACs).

In the present study we analyzed data from the VIKI study to evaluate the comparative performance of IACs vs. AACs calcifications in predicting VF, with the hypothesis that IACs correlate better than AACs with the presence of VF.

## 2. Materials and Methods

We conducted a secondary analysis of the VIKI study database. The VIKI study is a cross-sectional study involving 387 hemodialysis patients from 18 Italian dialysis centers [19]. Ethics committees approved the study (approval dates from 14 July 2008 to 26 October 2009) in accordance with regulations of observational studies. Patient enrollment, including adult patients who had been on hemodialysis for at least one year, took place between November 2008 and November 2009, and a follow-up to assess vital status was performed in December 2011. All participants signed an informed consent form to allow the use of their medical records for the study. We excluded patients with a life expectancy <6 months, with any evidence of cancer (except for basaliomas) or coagulation disorders, or who had any condition that, according to the investigator, might interfere with the study outcome. We collected the following information: anonymized demographic data (including gender and age); renal failure history (cause, type and duration of hemodialysis, history of transplantation); lifestyle data (smoking status, alcohol consumption); medical history and body mass index (BMI).

### 2.1. Data Collection

Fasting venous blood samples were collected from patients prior to the dialysis session for routine bone biochemistry, including total alkaline phosphatase (ALP), albumin, C-reactive protein (CRP), aluminum, parathyroid hormone (PTH), 25-OH vitamin D [2 5(OH)D] lipid profile and vitamin K components total BGP; undercarboxylated (uc) BGP; total MGP and ucMGP. Vitamin K components were determined by a simple, sensitive, and selective reverse-phase high performance liquid chromatography developed for the simultaneous determination of vitamin K in human plasma, as described in the previous VIKI study report [19]. Vitamin K values were corrected for triglyceride levels because vitamin K is transported on triglyceride-rich lipoproteins and may be affected by lipid-lowering medications [24].

### 2.2. Laboratory Assays

#### 2.2.1. Parathyroidhormone (PTH)

The method for quantitative determination of PTH in serum was the automated LIAISON^®^ N-Tact^®^ PTH Assay 310910, a direct, two-site, sandwich-type chemiluminescence immunoassay (CLIA) carried out on the LIAISON^®^ (DiaSorin Inc., Stillwater, MN, USA) instrument. The analytical sensitivity was 1 pg/mL and the intra-assay and inter-assay coefficients of variation (CVs) were 3.7–6.3 and 3.5–5.3%, respectively.

#### 2.2.2. 25-OH Vitamin D

For the quantitative determination of total 25-OH vitamin D (both the D2 and D3 forms) in serum, we used the automated LIAISON^®^ 25 OH Vitamin D TOTAL Assay 310600, a direct competitive CLIA executed on the LIAISON (DiaSorin) instrument. The analytical sensitivity was <10 nmol/L, and the intra-assay CV was between 2.9% and 5.5%, while the inter-assay CV was 6.3–12.9%.

#### 2.2.3. Total BGP

The method for the quantitative determination of total BGP in serum was the automated LIAISON^®^ Osteocalcin Assay 310950 (DiaSorin), a direct, two-site, sandwich-type CLIA executed on the LIAISON^®^ (DiaSorin) instrument. The analytical sensitivity was <0.3 ng/mL and the intra-assay CV was 3–8%, while the inter-assay CV was 4–9%.

#### 2.2.4. Undercarboxylated BGP (ucBGP)

For quantitative determination of the ucBGP, we used the Glu-osteocalcin Enzyme Immuno Assay (EIA) Kit MK118 (Takara Bio Inc., Otsu, Shiga, Japan), a manual solid-phase EIA based on a sandwich method that utilizes two mouse anti-ucBGP monoclonal antibodies to detect ucBGP by a two-step procedure. One of the mouse monoclonal anti-ucBGPs antibodies is immobilized onto the microtiter plate and blocked against non-specific binding. Samples are added to each well and incubated. The second step is to wash the plate and to add the second anti-BGP antibody labeled with peroxidase (POD). The reaction between POD and its substrate (H_2_O_2_ and 3,3′, 5,5′ tetramethyl-benzidine) results in color development, with intensities proportional to the amount of ucBGP present. The analytical sensitivity was 0.25 ng/mL and the intra-assay and inter-assay CVs were 4.4–6.7 and 5.7–9.9%, respectively.

#### 2.2.5. Total Matrix GLA Protein (MGP)

The quantitative determination of MGP was performed using the Human MGP-Matrix Gla Protein Kit (BiomedicaMedizinprodukte GmbH & Co KG, Wien, Austria). This is a manual competitive enzyme-linked immunosorbent assay (ELISA) method designed to detect MGP in serum. The analytical sensitivity was 0.3 nmol/L, and the intra-assay and inter-assay CVs were 5–6% and 7–9%, respectively.

#### 2.2.6. Undercarboxylated MGP (ucMGP)

The measurement of the total ucMGP was performed by the Vita K method [25], using a competitive ELISA, as described previously. The analytical sensitivity was 21 nmol/L, and the intra-assay and inter-assay CVs were found to be 8.9% and 11.4%, respectively.

### 2.3. Assessment of Vertebral Fracture and Vascular Calcification

The presence of VF and VC was determined from spine radiograms taken in the sagittal projection. According to the indications of Genant et al. [26], VF was defined as a deformity of the vertebral body due to reduction in one of its dimensions by more than 20%. Using a dedicated software (MorphoXpress^®^), all radiograms were assessed with quantitative vertebral morphometry (QVM), estimating VF severity as mild, moderate or severe (classified as height reduction: 20–25%, 25–40%, or >40%, respectively). Abdominal aorta calcifications (AACs) were quantified by measuring the length of calcific deposits along the abdominal aortic wall (graded as mild 0.1–5 cm, moderate 5.1–10 cm, or severe 10 cm), as described and validated previously [27]. IACs were evaluated from the same radiographic image and graded according to their length, as follows: mild 0.1–3 cm, moderate 3.1–5 cm, and severe >5 cm [19].

### 2.4. Statistical Analysis

Data are summarized as mean ± standard deviation (SD) for normally distributed variables, as median and interquartile range (IQ) for non-normally distributed variables; and as percentages for all categorical variables. The normal distribution of continuous variables was tested with the Shapiro-Wilk test. Categorical variables were analyzed by the chi-squared (χ^2^) test or Fisher’s exact method, as appropriate. The comparison between non-normally distributed continuous variables was performed by the Mann–Whitney rank test, whereas normally distributed continuous variables were compared by the unpaired Student’s t-test. The identification of correlates of VF was performed by univariate logistic regression analyses. All factors which correlated with VF with *p* < 0.10 at univariate analyses were jointly introduced into the same multivariable model. In these models, data were expressed as odds ratio (OR), 95% CI and *p* value. All statistical analyses were performed using the SPSS 15.0 statistical package. A value of *p* < 0.05 was considered statistically significant.

## 3. Results

The main demographic and clinical characteristics of the study population are summarized in Table 1. In 387 adult patients, IACs prevalence was 56.1% (N = 217). Patients with IACs were significantly older (median value: 70 vs. 63 years, *p* < 0.001) and presented a higher proportion of background cardiovascular comorbidities, such as myocardial infarction (23%, n = 50), atrial fibrillation (18.4%, n = 40) and peripheral vascular disease (40.6%, n = 88) as compared to those without. In the same patients, a higher prevalence of VF (64.1% vs. 44.1%, *p* < 0.001) was observed (Table 1). No significant differences were observed as to BMI, smoking or alcohol intake (Table 1). No significant between-groups differences were found in the levels of routine biochemical markers except for total cholesterol and triglycerides, which were slightly higher in patients with IACs (170.0 vs. 157.5, mg/dL and 155.5 vs. 134.0 mg/dL). A significantly lower median value of BGP total levels was observed in patients with IACs than in those without (164 vs. 206, mcg/L, *p* < 0.001). No differences were observed in MGP levels between the two groups (Table 1).

### 3.1. Interrelationship between Aortic and Iliac Calcifications

**Figure 1:**
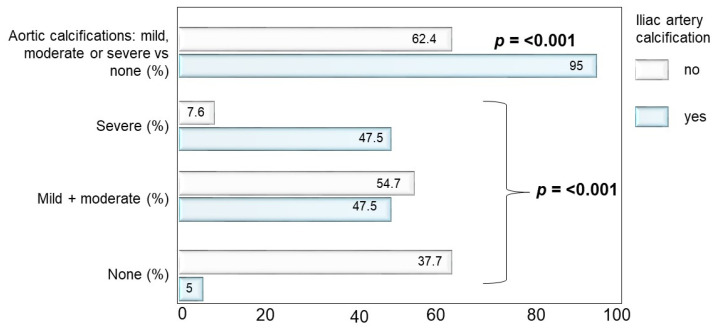
Proportion of patients with abdominal aorta calcifications (AACs) and frequency of AACs degree severity according to iliac arteries calcifications (IACs) presence (YES/NO). The prevalence of AACs as well as of severe AACs was significantly higher (*p* < 0.001) in patients with IACs than without.

**Figure 2:**
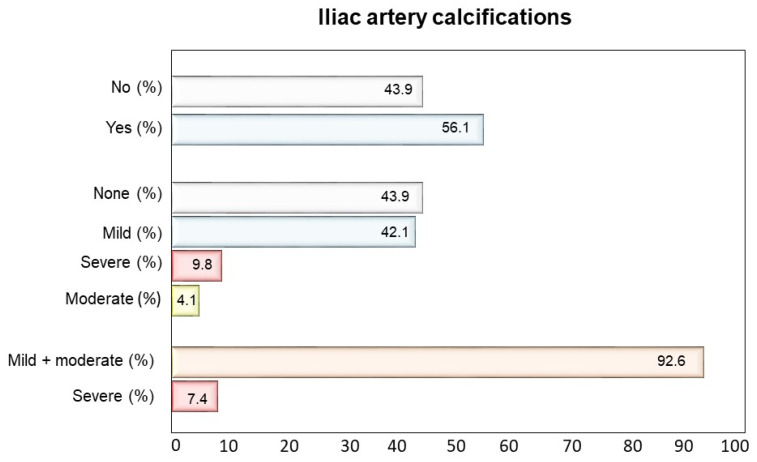
Proportion of patients with iliac arteries calcifications (IACs) and frequency of IACs degree severity.

The prevalence of AACs as well as of severe AACs was significantly higher (*p* < 0.001) in patients with IACs compared to those without (95.0% vs. 62.4% and 47.5% vs. 7.6%, respectively) (Figure 1). As for the severity of IACs, most patients had mild or moderate calcifications (92.6%) and only a small proportion had severe calcifications (7.4%) (Figure 2).

### 3.2. Vitamin K Levels and Pharmacological Treatment According to Iliac Artery Calcifications

**Figure 3:**
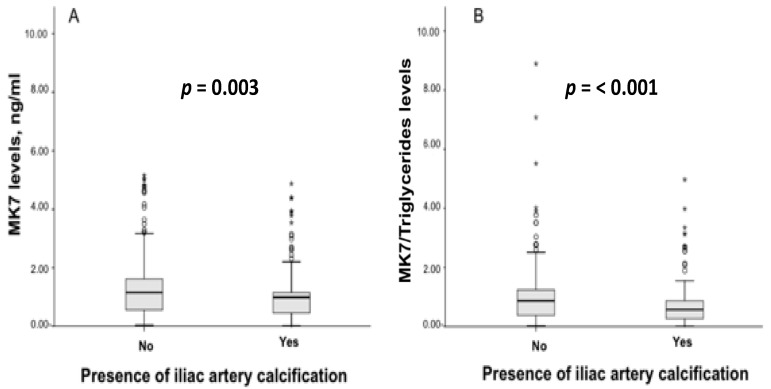
Levels of MK7 (**A**) and of MK7/Triglycerides (**B**) in hemodialysis patients with and without iliac artery calcifications (IACs). Data are presented as median 25th/75th percentiles and maximum/minimum recorded values. *p*-Values indicate statistically significant differences between patients with IACs compared to without. * Extreme value marked with star.

Circulating levels of MK7 were significantly lower in patients with IACs (0.99 vs. 1.15, ng/mL; *p* = 0.003) and this deficiency was magnified when MK7 levels were adjusted for triglycerides (0.57 vs. 0.87, ng/mL; *p* < 0.001). No significant differences between groups were observed for K1 and MK4 (Figure 3 and Supplementary Table S1). Some drug treatments, such as warfarin (17.1% vs. 5.3%, *p* = 0.001) and proton pump inhibitors (79.7% vs. 70.6%, *p* = 0.038) were more frequently prescribed in patients with IACs. (Supplementary Table S2).

### 3.3. Multiple Logistic Regression Model of Vertebral Fractures

**Figure 4:**
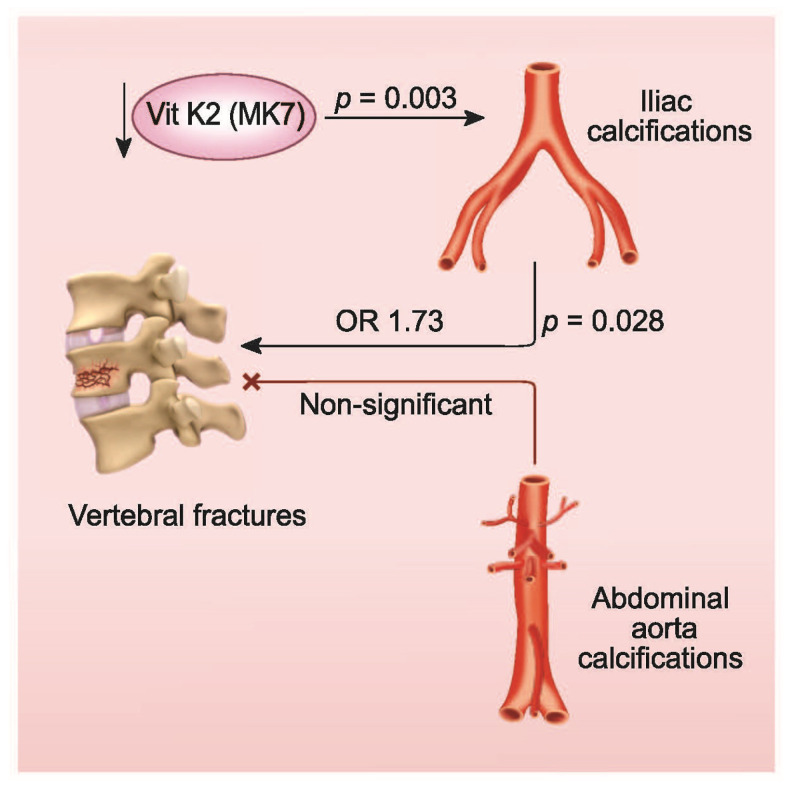
Iliac arteries calcifications (IACs) were associated in this study with 73% higher odds of VF (*p* = 0.028) whereas AACs were not (*p* = 0.294). Patients with IACs had lower levels of the vitamin K2, MK7 (0.99 vs. 1.15 ng/mL; *p* = 0.003).

A multivariable logistic regression analysis, adjusting for a series of potential confounders, showed that the presence of IACs was the third factor in rank order, after K1 deficit and sex, explaining the presence of VF (OR: 1.73, 95% CI 1.06–2.818; *p* = 0.028) (Table 2). Indeed, the OR of fractures was 73% higher in patients with IACs compared to those without. In the same model, age and vitamin D treatment were also significantly associated with the presence of vertebral fractures, while AACs failed to achieve significance in the association with the outcome variable (OR: 1.398, 95% CI: 0.747–2.617, *p* = 0.294) (Figure 4).

## 4. Discussion

In the general aging population, bone fractures are closely related to VC, and the association with AACs appears stronger for VF than for other fractures [11,28]. Our main result here confirms the association of VF and VC in hemodialysis patients, and sheds light on the importance of assessing IACs, rather than the more frequently evaluated AACs. In fact, in this secondary analysis of the VIKI study, we show, through a multivariable logistic regression analysis adjusted for a series of potential confounding variables, that the presence of IACs is a relevant factor explaining the presence of VF (OR:1.73), while AACs failed to associate with VF in the same model.

An insight into our results brings further evidence of the superiority of IACs over AACs in correlating with VF. We report a consistent co-presence of AACs and IACs (95% AACs prevalence in IACs-positive subjects) (Figure 1). However, non-severe AACs may be present in patients without IACs (62% non-severe AACs prevalence in IACs-negative subjects). Yet, VF are more strongly associated with IACs, suggesting a higher specificity of IACs. Remarkably, IACs associate with VF irrespective of calcification severity; in fact, IACs were mild or moderate in most subjects. In practical terms, the presence of IACs on a radiogram should alert physicians to the simultaneous presence or development of VF, avoiding the need for stratifying severe cases using debated thresholds, as usually done with AACs. This speculation would need to be tested in future prospective studies.

Our main results apparently contradict the findings by Rodriguez-Garcia et al. [29], who reported the association of medium-size (femoral, uterine/spermatic and radial), but not large-size arteries (the aorta and iliac arteries) with VF. However, the smaller sample size and the merging of AACs and IACs into one single, non-quantitative score may have hindered the role of IACs in that study. To our knowledge, our study is the first in hemodialysis patients that relates bone outcomes specifically to IACs.

The literature about the relationship between IACs and bone health is scarce, but is in line with our findings overall. One study on type 2 diabetes showed that IACs—but not AACs– are associated with an osteoporotic status [30]. One postmortem CT study found statistically significant differences only for IACs when comparing osteoporotic with non-osteoporotic subjects [31]. Another CT study confirmed an inverse association of lumbar bone mineral density with IACs, but the association was weaker than that for AACs [32]. However, that study was conducted in a subsample of the general population that was relatively young (aged 56 ± 11 years) and free from clinical cardiovascular disease, thus possibly less prone to the development of the medial calcifications common in iliac arteries [33]. It is documented that the wall of muscular or predominantly muscular arteries, such as the iliac and femoral arteries, is more susceptible to develop calcification [20,21,22].This could be related to the higher density of smooth muscle cells present in the wall of these vessels, which are those that have eventually undergone bone- and cartilage-like phenotypic changes under several in vitro experimental conditions [33,34]. Our results indirectly corroborate this hypothesis.

In terms of survival of hemodialysis patients, both bone fractures [35], VF in particular [29], and IACs [36] have already been reported as negative prognostic factors. Our study, now suggesting the strong association of IACs to VF, leads us to hypothesize that the timely prediction of VF through detection of IACs by the adoption of proper preventive measures might indirectly lead to the extended survival of CKD patients. In addition, Kwon et al. recently reported, in a large hemodialysis cohort, of the association between VF (but not other fractures) with myocardial infarction [10]. Our results indeed suggest the relationship of IACs with poor cardiovascular health (e.g., myocardial infarction, atrial fibrillation and peripheral vascular disease, as reported in Table 1). Thus, we can speculate that, if the link is causal, which cannot be excluded, the prompt identification of VFs has a potential to reduce the incidence of cardiovascular comorbidities in hemodialysis patients.

Another specific finding of our study is that the deficit of the menaquinone-7 (MK-7) is a predictor of IACs: patients with IACs featured significantly lower circulating levels of MK7 than patients without, and this deficiency was greater when MK7 levels were adjusted for triglycerides. MK7 has shown unique characteristics in terms of bioavailability and biological effects, and is superior to other components of the vitamin K family [37]. The Rotterdam study was one of the first that reported a protective role of menaquinone intake against incident coronary heart disease and related mortality. Moreover, the OR of severe AACs was significantly lower in patients with the highest menaquinone intake compared with those with the lowest intake [38]. The role of vitamin K in VC is mainly related to the action of VKDP such as MGP, which is released from vascular smooth muscle cells and chondrocytes. The potential mechanism through which MGP prevents VC could be due to the interaction with BMP-2, a powerful osteo-inductive protein of the TGF-beta family, transforming undifferentiated cells and subpopulations of vascular smooth muscle cells into ostoblast-like cells [39].

Our study has strength in that the link between IACs and VF remained significant in a model adjusting for a series of potential confounders, suggesting an independent role of this risk factor in explaining the high frequency of VF in the study population. Our findings also suggest that IACs may be a clinically relevant tool to be used as a risk factor for VFs regardless of calcification severity, thus possibly helping adverse cardiovascular events and perhaps mortality. All of this is enhanced by the association between IACs and vitamin K, the latter being known to be a main player in bone and vascular health. Prospective studies are needed to confirm these findings both in the general population and in patients with chronic kidney disease.

This study also has important limitations. The main one is its cross-sectional nature, which makes it unable to delineate a time sequence and even more to infer causality relationships. Nonetheless, being the first of its kind in this patient population, this study has to be recognized as a pioneering and hypothesis-generating one.

In conclusion, based on our findings, clinicians should pay particular attention to patients with IACs because such patients may be at high risk of VFs, regardless of their severity. As a hypothesis, early prevention measures triggered by the detection of VF may prevent cardiovascular adverse events. This proposition is supported by the association between IACs and vitamin K, which has a key role in both bone and vascular health. Prospective studies are needed to confirm these remarkable findings both in the general population and in patients with chronic kidney disease.

## Figures and Tables

**Table 1:** Main demographic and clinical characteristics of the patients.

Variable		Iliac Calcification (Yes)	Iliac Calcification (No)	*p*-Value
		N = 217, 56.1%	N = 170, 43.9%	
Demographic variables and risk factors				
Sex, female, n (%)		79 (36.4%)	66(38.8%)	0.626
Age, years		70 (61.5, 76)	63 (47, 72)	**<0.001**
Weight, kg		70.53 ± 13.56	69.74 ± 15.83	0.601
Height, meters		1.67 (1.60, 1.73)	1.68 (1.60. 1.75)	0.633
BMI, kg/cm^2^		24.68 (22.39, 28.12)	24.2 (21.37. 27.73)	0.157
Smoker, n (%) (n = 370)				0.475
	Yes	24 (11.8%)	27 (16.2%)	
	No	132 (65.0%)	102 (61.0%)	
	Ex	47 (23.2%)	38 (22.8%)	
Current or former alcohol drinker		48 (23.8%)	34 (21.4%)	0.592
n (%) (n = 361)				
Medical history				
Dialysis vintage, months, median		49 (29.5, 101.5)	49.5 (26, 85)	0.452
Type of dialysis, n (%)				0.577
	Bicarbonate dialysis	113 (52.1%)	76 (44.7%)	
	Hemofiltration (HF)	17 (7.8%)	15 (8.8%)	
	Hemodiafiltration (HDF)	51 (23.5%)	51 (30.1%)	
	Acetate free biofiltration (AFB)	31 (14.3%)	23 (13.5%)	
	Other types of dialysis	5 (2.3%)	5 (2.9%)	
Previous kidney transplant, n (%)		39 (13.8%)	24 (14.1%)	0.934
Hypertension, n (%)		168(77.4%)	136 (80%)	0.539
Angina, n (%)		40 (18.4%)	24 (14.1%)	0.257
Myocardial infarction, n (%)		50 (23.0%)	23 (13.5%)	**0.018**
Atrial fibrillation, n (%)		40(18.4%)	11 (6.5%)	**0.001**
Heart failure, n (%)		26 (12.0%)	13 (7.6%)	0.16
Diabetes mellitus, n (%)		54 (24.9%)	31 (18.2%)	0.117
Peripheral vascular disease, n (%)				**0.014**
	No	129 (59.4%)	124 (72.9%)	
	Asymptomatic	65 (30.0%)	33 (19.4%)	
	Intermittent claudication	20 (9.2%)	8 (4.8%)	
	Amputation	3 (1.4%)	5 (2.9%)	
Cerebrovascular accident, n (%)				0.209
	No	191 (88.0%)	155 (91.2%)	
	Stroke	15 (6.9%)	5 (2.9%)	
	Other type	11 (5.1%)	10 (5.9%)	
Vertebral fractures, n (%)		139 (64.1%)	75 (44.1%)	**<0.001**
Routine biochemical profile				
Ca, mg/dL		9.20 ± 0.71	9.10 ± 0.64	0.141
P, mg/dL		4.72 ± 1.32	4.83 ± 1.21	0.366
Mg, mg/dL (n = 139)		2.40 ± 0.56 (n = 61)	2.44 ± 0.59 (n = 78)	0.644
Alkaline phosphatase, U/L		85 (65, 111)	80 (63, 110)	
PTH, pg/mL		240 (134, 384)	244 (143. 379)	
Albumin, d/dL		3.81 ± 0.49	3.85 ± 0.40	
CRP, mg/L		1.9 (0.58, 5.50)	1.02 (0.38. 4.50)	
KT/V		1.24 ± 0.26	1.26 ± 0.28	
Aluminium, mcg/L		(n = 60)13.6 (8.5, 22.0)	(n = 107) 10.2 (7.2, 17.8)	
Total cholesterol, mg/dL		170 (146.25, 194)	157.5 (131.75, 191.25)	**0.025**
Tryglicerides, mg/dL		155.5 (116.5, 225)	134(100, 185)	**0.002**
HDL Cholesterol, mg/dL		40.00 (32.00, 49.25)	40 (33, 50)	0.981
LDL Cholesterol, mg/dL		93 (70.75, 116)	89 (66, 118)	0.455
25(OH) vitamin D, ng/mL		28.8 (19.25, 43.85)	28.95 (19.08, 46.14)	0.382
BGP total, mcg/L		164 (84.65, 266.5)	206 (106, 373.75)	**0.011**
BGP undecarboxylated, ng/mL		10.08 (4.31, 17.10)	12.03 (4.69, 18.65)	0.148
MGP total, nmol/L		18.67 (12.13, 30.34)	18.97 (13.20, 31.64)	0.598
MGP decarboxylated, nmol/L		583 (318, 1030)	533 (259, 878)	0.183

Data are given as n (%), mean and standard deviation or as median and interquartile range, as appropriate. BGP, bone Gla protein; BMI: Body Mass Index, 25(OH)D, 25-hydroxyvitamin D or calcifediol; Ca, calcium; CRP, C-reactive protein; HDL, high-density lipoprotein; LDL, low-density lipoprotein; Mg, magnesium; MGP, matrix Gla protein; P, phosphorus; PTH, parathyroid hormone; SD, standard deviation. In bold the statistically significant *p*-value.

**Table 2:** Multiple Logistic Regression with the presence of fractures as outcome *.

Variables *	Odds Ratio	95% CI	*p* Value
Deficit of vitamin K1 (yes/no)	2.929	(1.324, 6.482)	**0.008**
Gender (female vs. male)	0.533	(0.332, 0.854)	**0.009**
Iliac Calcifications (yes/no)	1.73	(1.060, 2.818)	**0.028**
Age (years)	1.02	(1.00, 1.04)	**0.043**
Oral Calcitriol (yes/no)	0.598	(0.363, 0.985)	**0.043**

* adjusted for age; albumin, alkaline phosphatase, aortic calcifications, cholesterol, deficit of menaquinone-4/triglycerides, deficit of vitamin K1, iliac artery calcifications, myocardial infarction, parathyroidhormone, total matrix-Gla-protein, total bone-Gla-protein, sex and the use of the following therapies: aluminium, calcimimetics, calcium acetate, lanthanum, oral calcitriol, proton pomp inhibitors, sevelamer, vitamin D analogues, i.v. vitamin D. Only variables achieving statistical significance are shown. In bold the statistically significant *p*-value.

## Data Availability

We exclude this statement.

## Supplementary Materials

The following are available online at https://www.mdpi.com/article/10.3390/nu13103567/s1, Table S1: Vitamin K Status by presence of iliac artery calcifications, Table S2: Therapy by the presence of iliac artery calcifications.
