# Supplementary material for: The Vessels-Bone Axis: Iliac Artery Calcifications, Vertebral Fractures and Vitamin K from VIKI Study
Source: Nutrients. 2021 Oct 12;13(10):3567. doi: 10.3390/nu13103567 (PMC8539275; doi:10.3390/nu13103567)
Supplement: Supplementary file 1 [file nutrients-13-03567-s001.zip › nutrients-1371827-supplementary.pdf]

Supplementary Table S1: Vitamin K Status by presence of iliac artery calcifications

Vitamers	Iliac Calcification (Yes) N=217, 56.1%	Iliac Calcification (No) N=170, 43.9%	P-value
K1, ng/mL	0.62 (0.31, 1.06)	0.64 (0.35, 1.07)	0,695
K1/triglycerides,	0.40 (0.22, 0.70)	0.51 (0.23, 0.82)	0,116
MK4 ng/mL	0.56 (0.18, 0.67)	0.49 (0.25, 0.67)	0,526
MK4/triglycerides	0.35 (0.12, 0.51)	0.41 (0.15, 0.51)	0,19
MK7 ng/mL	0.99 (0.44, 1.15)	1.15 (0.56, 1.63)	**0,003**
MK7/triglycerides	0.57 (0.26, 0.87)	0.87 (0.38, 1.26)	**<0.001**

Data are given as median and interquartile range. Abbreviations: K1, vitamin K1; MK, menaquinone.

Supplementary Table S2. Therapy by the presence of iliac artery calcifications

Drugs prescribed	Iliac Artery Calcifications (Yes) N=217, 56.1%	Iliac Artery Calcifications (No) N=170, 43.9%	P-value
Warfarin (n, %)	37 (17.1%)	9 (5.3%)	**0.001**
Steroids (n, %)	12 (5.5%)	9 (5.3%)	0.919
Thyroid hormones (n, %)	22 (10.1%)	18 (10.6%)	0.885
Antibiotics (n, %)	10 (4.6%)	6 (3.5%)	0.597
Anti-epileptic drugs (n, %)	10 (4.6 %)	4 (2.4 %)	0.238
Statins (n, %)	72 (33.2%)	54 (31.8%)	0.768
Beta-blockers (n, %)	80 (36.9%)	64 (37.6%)	0.875
Non-insulin glucose-lowering drugs	3 (1.4%)	4 (2.4 %)	0.477
Insulin (n, %)	37 (17.1 %)	21 (12.4 %)	0.199
PPI (n, %)	173 (79.7%)	120 (70.6%)	**0.038**
Aluminium (n, %)	57 (26.3%)	39 (22.9%)	0.452
Calcium Carbonate (n, %)	78 (35.9%)	54 (31.8%)	0.389
Calcium Acetate (n, %)	10 (4.6 %)	11 (6.5 %)	0.422
Sevelamer (n, %)	90 (41.5%)	73 (42.9%)	0.772
Lanthanum (n, %)	26 (12.0 %)	30 (17.6 %)	0.116
Intravenous calcitriol (n, %)	8 (3.7 %)	4 (2.4 %)	0.453
Oral calcitriol (n, %)	92 (42.4 %)	85 (50.0 %)	0.136
Vitamin D analogs (n, %)	46 (21.2 %)	31 (18.2 %)	0.469
Calcimimetics (n, %)	37 (17.1 %)	38 (22.4 %)	0.19

Abbreviations: PPI, proton pump inhibitors.
